# A comparative analysis of the binary and multiclass classified chest X-ray images of pneumonia and COVID-19 with ML and DL models

**DOI:** 10.1515/med-2024-1110

**Published:** 2025-02-04

**Authors:** Madhumita Pal, Ranjan K. Mohapatra, Ashish K. Sarangi, Alok Ranjan Sahu, Snehasish Mishra, Alok Patel, Sushil Kumar Bhoi, Ashraf Y. Elnaggar, Islam H. El Azab, Mohammed Alissa, Salah M. El-Bahy

**Affiliations:** Department of Electrical Engineering, Government College of Engineering, Keonjhar, Odisha, India; Department of Chemistry, Government College of Engineering, Keonjhar, 758 002, Odisha, India; Department of Chemistry, School of Applied Sciences, Centurion University of Technology and Management, Balangir, Odisha, India; Department of Botany, Vikash Degree College, Barahaguda, Canal Chowk, Bargarh, Odisha, India; School of Biotechnology, Campus-11, KIIT Deemed-to-be-University, Bhubaneswar, Odisha, India; Department of Civil Engineering, Government College of Engineering, Keonjhar, Odisha, India; Department of Electrical Engineering, Government College of Engineering Kalahandi, Kalahandi, Bhawanipatna, 766 003, Odisha, India; Department of Food Sciences and Nutrition, College of Science, Taif University, Taif, Saudi Arabia; Department of Medical Laboratory, College of Applied Medical Sciences, Prince Sattam bin Abdulaziz University, Al-Kharj, Saudi Arabia; Department of Chemistry, Turabah University College, Taif University, Taif, Saudi Arabia

**Keywords:** COVID-19, chest X-ray images, ML and DL models, binary and multiclass classification

## Abstract

**Background:**

The highly infectious coronavirus disease 2019 (COVID-19) is caused by severe acute respiratory syndrome coronavirus 2, the seventh coronavirus. It is the longest pandemic in recorded history worldwide. Many countries are still reporting COVID-19 cases even in the fifth year of its emergence.

**Objective:**

The performance of various machine learning (ML) and deep learning (DL) models was studied for image-based classification of the lungs infected with COVID-19, pneumonia (viral and bacterial), and normal cases from the chest X-rays (CXRs).

**Methods:**

The *K*-nearest neighbour and logistics regression as the two ML models, and Visual Geometry Group-19, Vision transformer, and ConvMixer as the three DL models were included in the investigation to compare the brevity of the detection and classification of the cases.

**Results:**

Among the investigated models, ConvMixer returned the best result in terms of accuracy, recall, precision, *F*1-score and area under the curve for both binary as well as multiclass classification. The pre-trained ConvMixer model outperformed the other four models in classifying. As per the performance observations, there was 97.1% accuracy for normal and COVID-19 + pneumonia-infected lungs, 98% accuracy for normal and COVID-19 infected lungs, 82% accuracy for normal + bacterial + viral infected lungs, and 98% accuracy for normal + pneumonia infected lungs. The DL models performed better than the ML models for binary and multiclass classification. The performance of these studied models was tried on other CXR image databases.

**Conclusion:**

The suggested network effectively detected COVID-19 and different types of pneumonia by using CXR imagery. This could help medical sciences for timely and accurate diagnoses of the cases through bioimaging technology and the use of high-end bioinformatics tools.

## 1 Introduction

Coronavirus disease 2019 (COVID-19) is a highly infectious disease caused by severe acute respiratory syndrome coronavirus (SARS-CoV-2), a coronavirus strain [1,2]. First reported in December 2019 from Wuhan City, China, the scenario became global, for which the World Health Organisation (WHO) officially declared the outbreak as a pandemic in March 2020 [3]. Several COVID-19 waves ensued globally as multiple viral variants emerged, particularly the variants of concern that affected almost every region around the globe [4,5]. A total of 776,696,616 COVID-19 cases with 7,072,509 deaths were reported globally as of 20 October 2024 (https://data.who.int/dashboards/covid19/cases?n=o). Some countries are still reporting COVID-19 infection cases even in the fifth year of the first report of human infection by the virus [6]. The pandemic reshaped the global economy, community health, family life, jobs and employment, education, and social and cultural ethos [7,8]. The impact varied between countries at the global level as this pandemic triggered the largest global economic crisis ever, with increasing poverty and inequalities [9,10]. Further, it presented unprecedented pressure on the health infrastructure that challenged public health and diminished livelihood options, food security, and nutrition provisions, especially in low-income countries with less purchasing capacity [11].

Foolproof diagnostic options are essential tools for swift pandemic response and to activate community health measures. The three widely used tests for diagnosis to detect COVID-19 cases are molecular (PCR), antigen rapid detection, and antibody-based [12]. PCR (polymerase chain reaction) is highly sensitive and specific in detecting RNA viruses. PCR is recommended by the WHO, especially to confirm symptomatic cases. Although antigen-based rapid test detects viral proteins; however, the test is less sensitive compared to molecular tests. The antibody-based test detects the antibody titre in the subject as the response to infection or vaccination and could be a useful public health surveillance tool. These three diagnosis approaches played a crucial role in transitioning from pandemic response to pandemic control. Among these, PCR (especially, reverse transcription polymerase chain reaction; RT-PCR) is the most reliable technique and is widely being employed in diagnosing COVID-19 since the outbreak began [13,14]. Although RT-PCR sensitively and quantitatively detects SARS-CoV-2, it needs skilled clinical laboratory staff with a complicated procedure that costs US$50–100 per test on average, which may not be suitable for low-income countries [15]. Many countries lack adequate supply of RT-PCR test kits, and hence, an alternative automatic diagnostic system for early detection of COVID-19 to prevent its further spread is essential.

Pneumonia is a frequently encountered lung infection, being severe in the elderly and children below 5 years. It hinders a patient’s oxygen intake ability through the lungs into the bloodstream. Pneumonia could be detected by employing numerous biotechniques like chest X-ray (CXR), computer-aided tomography, and magnetic resonance imaging. These need expert radiologists for the purpose, which could often lead to delayed or misdiagnosis. Similar to influenza, clinical investigations have confirmed that COVID-19 affects the lower part of the respiratory tract, especially the lungs [16]. The computed tomography (CT) scan of the chest is an effective imaging technique to diagnose lung-related diseases. However, CXR is a widely accepted alternative in hospitals due to its faster imaging time and low cost compared to the CT scan [16,17]. Thus, CXR images could be very useful in the early diagnosis of COVID-19 and probably could help in prediagnosis and prodiagnosis, too.

Radiologists frequently struggle to distinguish COVID-19 from other pulmonary conditions and community-acquired pneumonia based simply on the X-ray images and CT scans [18]. To promptly confirm COVID-19 cases, the artificial intelligence (AI) approach seems to be appealing to researchers and physicians owing to the expected high accuracy and a lower operational cost [19]. Researchers have employed the open-source COVID-19 database to gather and evaluate radiography data. The digitised X-ray image versions are typically used to classify automatically using machine or deep learning (DL) tools alongside traditional image processing tools [20]. AI technologies are promising in resolving numerous issues with the aid of novel machine learning (ML) and DL tools. It includes enhanced access to high-quality healthcare, especially in rural and low-income areas; resolving the skewed issue of the number of patients and skilled physicians; enhancing the efficiency and training of the healthcare personnel especially engaged in complex procedures; and facilitating the large-scale delivery of personalised healthcare.

Given the scenario, ML- and DL-based computer-aided diagnostic approaches are significant to timely and accurately detect COVID-19 and pneumonia. As the X-ray technique is cheaper compared to other techniques, it was chosen in this investigation as base image data. Pneumonia and COVID-19 detection by using ML or DL techniques have been reported. However, comparative studies on pneumonia and COVID-19 detection using ML and DL simultaneously and comparing the performance are few. This study compared the performance of ML and DL techniques to diagnose COVID-19 and pneumonia using CXR images. Two ML models (the *K*-nearest neighbour (*K*-NN) and logistics regression (LR)) and three DL models (Visual Geometry Group-19 (VGG-19), Vision transformer (ViT) and ConvMixer) were chosen to classify pneumonia and COVID-19 CXR images, with images of healthy individuals as the control. The study was conducted with the following as the primary objectives:

- To implement ML/DL model for binary and multiclass classification of CXR images of normal, COVID-19, and pneumonia cases
- To differentiate COVID-19-infected lung images from pneumonia and normal CXR images
- To classify and differentiate pneumonia subtypes from CXR images using ML/DL models
- To compare the performance of ML and DL models in terms of accuracy, recall, precision, *F*1-score, and area under the curve (AUC)
- To compare the performance of the proposed models with other reported models

## 2 Methodology

The preparatory steps that were carried out for CXR image classification were data collection, image preprocessing to reduce the “noise,” implementation of ML/DL models, and measuring the performance of the models in terms of accuracy, recall, precision, *F*1-score, and AUC.

### 2.1 Data collection

Two datasets were chosen for the investigative study. The CXR image dataset was sourced from https://data.mendeley.com/datasets/rscbjbr9sj/2. This dataset contained 5,232 X-ray images of paediatric (1–5 years) cases from Guangzhou Medical Centre, China. It contained X-ray images of the normal chest, the bacterial pneumonia-infected chest, and the viral pneumonia-infected chest. Out of the total 5,856 images in the dataset, 1,583 were normal, and 4,273 were pneumonia cases, of which 2,538 were bacterial pneumonia, and 1,735 were viral pneumonia cases. The second dataset was collected from https://github.com/ieee8023/covid-chestxray-dataset. It consisted of 10,192 normal images, 3,616 COVID-19-positive cases, 6,012 lung opacity (non-COVID lung infection), and 1,345 viral pneumonia images. As the dataset collected from these two public repositories was not balanced, the synthetic minority oversampling technique was used to balance them; 80% of the data was used to train the models, and 20% was used to test the models.

### 2.2 Image pre-processing

Pre-processing is the first and critical step wherein the images are moderated, and quality is enhanced to improve the performance of computer-assisted diagnosis. Here, the necessary conversion of the raw input images into the correct format is done before feeding to the DL classifiers. This step is very important as the clinical datasets available online to feed the network classifiers often had images of varying size, shape, and contrast. The images ought to be of the same size as the network classifier’s input before feeding. Thus, the images need rescaling or resizing to fit the intended input format.

During the image pre-processing phase, the quality of images was enhanced, and the “noise” was reduced by using contrast-limited adaptive histogram equalisation and numerous other data augmentation techniques like normalisation, resizing, horizontal random flip, random rotation, and zoom. Data augmentation techniques were implemented on the image database to address the over-fitting issue. Normalisation, resizing, horizontal random flip, random rotation with factor 0.2, and zoom (height factor = 0.2, width factor = 0.2) were the steps followed during augmentation.

### 2.3 Performance parameters employed to compare the ML/DL models

Two ML models (LR and *k*-NN) and three DL models (VGG19, ViT, and Convmixer) were implemented to detect and classify CXR images. The performance of these models was evaluated in terms of accuracy, recall, precision, *F*1-score, and AUC employing the following equations. The definition of each performance evaluation matrix is detailed in Table 1:

(1) \[\text{Precision}=\frac{\text{True}\hspace{.25em}\text{positive}}{\text{True}\hspace{.25em}\text{positive}+\text{False}\hspace{.25em}\text{positive}},]\]

(2) \[\text{Recall}=\frac{\text{True}\hspace{.25em}\text{positive}}{\text{True}\hspace{.25em}\text{positive}+\text{False}\hspace{.25em}\text{negative}},]\]

(3) \[F\text{1-score}=\frac{2\times \text{precision}\hspace{.25em}\times \hspace{.25em}\text{recall}}{\text{precision}\times \text{recall}},]\]

(4) \[\hspace{7em}\text{Accuracy}=\frac{\text{True}\hspace{.25em}\text{positive}+\text{True}\hspace{.25em}\text{negative}}{\text{True}\hspace{.25em}\text{positive}\hspace{.25em}+\hspace{.25em}\text{True}\hspace{.25em}\text{negative}+\text{False}\hspace{.25em}\text{positive}\hspace{.25em}+\hspace{.25em}\text{False}\hspace{.25em}\text{negative}}.]\]

**Table 1:** Various training parameters considered to train the models

Model	Training parameters
LR	Sigmoid function
*k*-NN	Neighbours = 7
ViT	Learning rate = 0.001
	Weight decay = 0.0001
	Batch size = 20
	Image size = 72
	Patch size = 6
	Transformer layer = 8
	MLP head units = [2,048, 1,024]
ConvMixer	Learning rate = 0.001
	Weight decay = 0.0001
	Loss = sparse categorical loss function
	Optimiser = Adam
	Batch size = 8
	Epoch = 50
	Image size = 150
	Filters = 256
	Depth = 8
	Kernel size = 5
	Number of classes = 3
	Global average pooling
	Activation = softmax
VGG-19	Relu activation function
	Batch size = 32
	Epoch = 50
	Trainable parameters = 20,024,384 (76.39 MB)

The AUC is the curve between the true-positive rate and the false-positive rate. AUC was used to compare the performances of two separate models using a roughly balanced dataset.

### 2.4 Description of the ML and DL models employed in the study

LR and *k*-NN as ML models, ViT, VGG-19, and ConvMixer models were used to detect COVID-19 and pneumonia infection from the CXR images. The training parameters used to test the models are detailed in Table 1, and the details of each of the five models are provided in separate sections below.

Total parameters: 20,024,384 (76.39 MB)

Trainable parameters: 20,024,384 (76.39 MB)

Non-trainable parameters: 0 (0.00 Byte)

#### 2.4.1 LR

**Figure 1:**
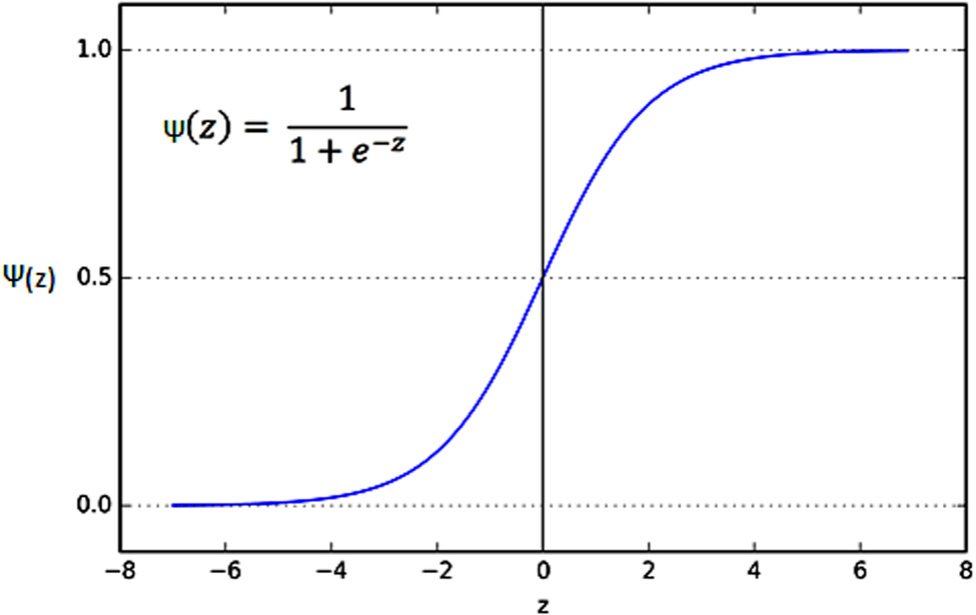
Output of sigmoid function for the predicted probability value.

LR is implemented for the linearly separable data. It is a supervised ML model [21] used for binary classification tasks. It provides discrete outcomes such as 0 and 1. It is used to predict the probability of occurrence of certain events or classes of events. Single independent variables or multiple independent variables are used to predict the outcome of logistic regression. The predicted probability value is converted into 1 or 0 using the sigmoid activation function [22] (Figure 1). The sigmoid function is provided in equation (5):

(5) \[\Psi (z)=\frac{1}{1+{e}^{-z}}.]\]

#### 2.4.2 *k*-NN

*k*-NN is the most popular and simple distance-based supervised ML algorithm used for regression as well as classification tasks, although commonly used for classification tasks. It is a lazy nonparametric learning model [23] as it learns during the testing phase and stores data only during the training phase. The *k*-NN model follows the nearest data point and classifies the data point accordingly [21]. Its classification is based on the consensus of its *k* neighbours. The case assigned to the class shares the most occurrences with its *k* nearest neighbour, as determined by a distance function. Different distance matrices like Manhattan distance, Euclidean distance, cosine distance, and Minkowski distance are used to ascertain the distance between all the training data and new data points. The *k* value could be properly chosen to get better classification results during the testing phase, or else the model either overfits (*k* = 1) or underfits (higher *k* value).

#### 2.4.3 ViT

**Figure 2:**
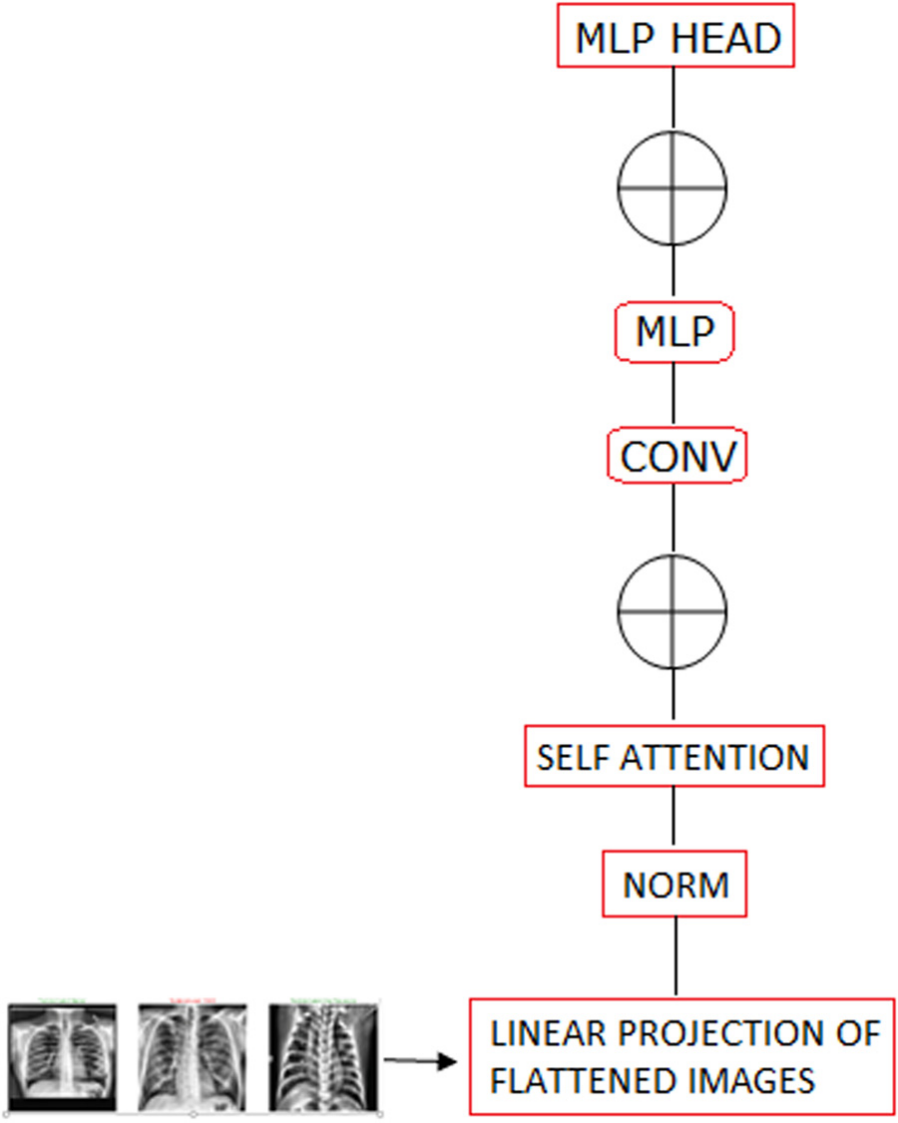
The structure of the ViT model.

An advanced DL architecture created, especially for visual recognition applications, is the ViT Model [24]. It is a novel model combining the capabilities of transformer models with computer vision that was originally developed for natural language processing. The self-attention mechanism at the core of the operational logic of the ViT Model allowed it to recognize contextual information and global interdependencies within an image. ViT Model leverages the attention mechanism [25] to directly extract relevant visual features from unprocessed pictures, unlike conventional convolutional neural networks (CNNs) that depend on hand-crafted features. An input image is divided into smaller patches by the ViT Model, treating them as consecutive tokens. These patches were then fed into a transformer encoder that has several layers of feed-forward and self-attention neural networks (Figure 2). The model can selectively focus on different parts of an image and understand the complex relationships through the self-attention mechanism, and the feed-forward networks process the attained data to produce meaningful visual embeddings. ViT Model learns to use large image datasets (like ImageNet with large labeled images) during the training phase. The model learns to predict the right class labels for the photos through supervised learning, fine-tuning its parameters using gradient descent and back-propagation techniques. In addition, the ViT Model presents notable interpretability and scalability benefits. It is capable of handling a wide range of image sizes and complexity levels with ease. By examining and explaining the decision-making process of the model, researchers and practitioners could better understand the variables influencing the model’s predictions, made possible by the self-attention mechanism.

In summary, the (Google) ViT Model uses the remarkable combined power of transformer models for visual identification tasks, epitomising a paradigm shift in computer vision. Its capacity to recognise global interdependencies and self-attention mechanisms has led to advancements in various application disciplines. The ViT Model has transformed computer vision by utilising large datasets and cutting-edge training techniques, opening up new avenues for investigations and useful applications.

#### 2.4.4 ConvMixer

**Figure 3:**
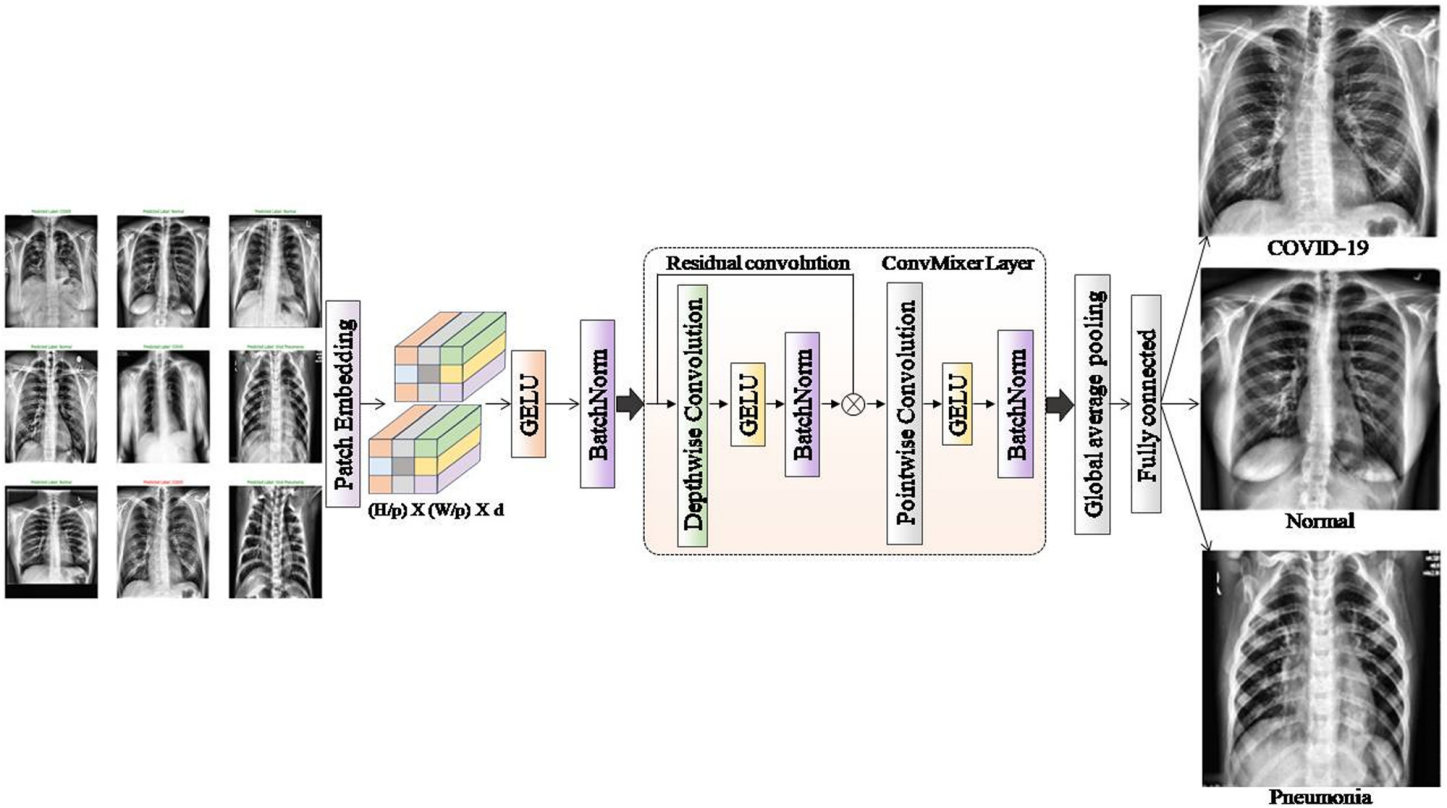
The architecture of the ConvMixer model.

ConvMixer bears many similarities to ViT (and MLP-Mixer); it operates directly on patches, preserves an equal-resolution-and-size representation across all layers [25], does not down-sample the representation at successive layers, and differentiates between “spatial mixing” and “channel-wise mixing” of information (Figure 3). ConvMixer performs all these using ordinary convolutions, unlike ViT and MLP-Mixer. ConvMixer consists of a patch-embedding layer and a simple, fully convolutional block applied repeatedly. Convolution with *c*_in_ input channels, *h* output channels, *p* kernel size, and *p* stride could be used to implement patch embeddings with *p* patch size and *h* embedding dimension (equation (6)).

(6) \[{z}_{0}=\text{BN}(\alpha \hspace{.5em}{\text{Conv}}_{\text{cin}\to h(X,\text{Stride}=p,\text{kernel}\text{size=}p}).]\]

#### 2.4.5 Visual geometric group-19 (VGG-19)

A VGG network is built using minuscule convolutional filters [26]. Thirteen convolutional layers and six fully connected layers make up the VGG-19 (that is, 19 layers deep). It consists of convolutional, hidden, and fully connected layers. The network structure of the VGG-19 model is shown in Table S1.

Quickly examining the VGG architecture, as the 224 × 224 picture is fed into the VGG Net (very deep convolutional network), the developers chop out the central 224 × 224 patch for each image for the ImageNet competition to maintain a uniform input size of the image. The convolutional layers of VGG make use of a minimal receptive field, i.e. 33, the least size that still captures left-right and up-down. Additionally, a 1X1 convolution filter is used to transform the input linearly. Rectified linear unit activation function and a significant AlexNet innovation that reduces training duration come next. ReLU gives an output if the input is positive, else it gives zero output. The convolution stride is fixed at one pixel to maintain the spatial resolution after convolution (stride is the number of pixel shifts over the input matrix). The VGG Net consists of three fully connected layers, each of which has 4,096 channels, and the third layer has 1,000 channels, one for each class.

### 2.5 The simulation software

Google Collaboratory, a “Cloud” platform-hosted free Jupyter notebook, was used to conduct the study. Free GPU access and a zero-configuration interface were also included to create and run Python code straight from the browser using well-known Python packages to aid in data analysis. The Colab was used for visualization purposes. The input datasets could be images and train classifiers to assess the classifiers’ performance. Tesla T4 device was used and the computing capability was 7.5.12.7 GB RAM used for the simulation job. Pci bus id: 0000:00:04.0. Cloud server was employed to use the GPU’s power.


**Ethical approval**: The nature of this article does not require any ethical approval.

## 3 Results of the simulation work

**Figure 4:**
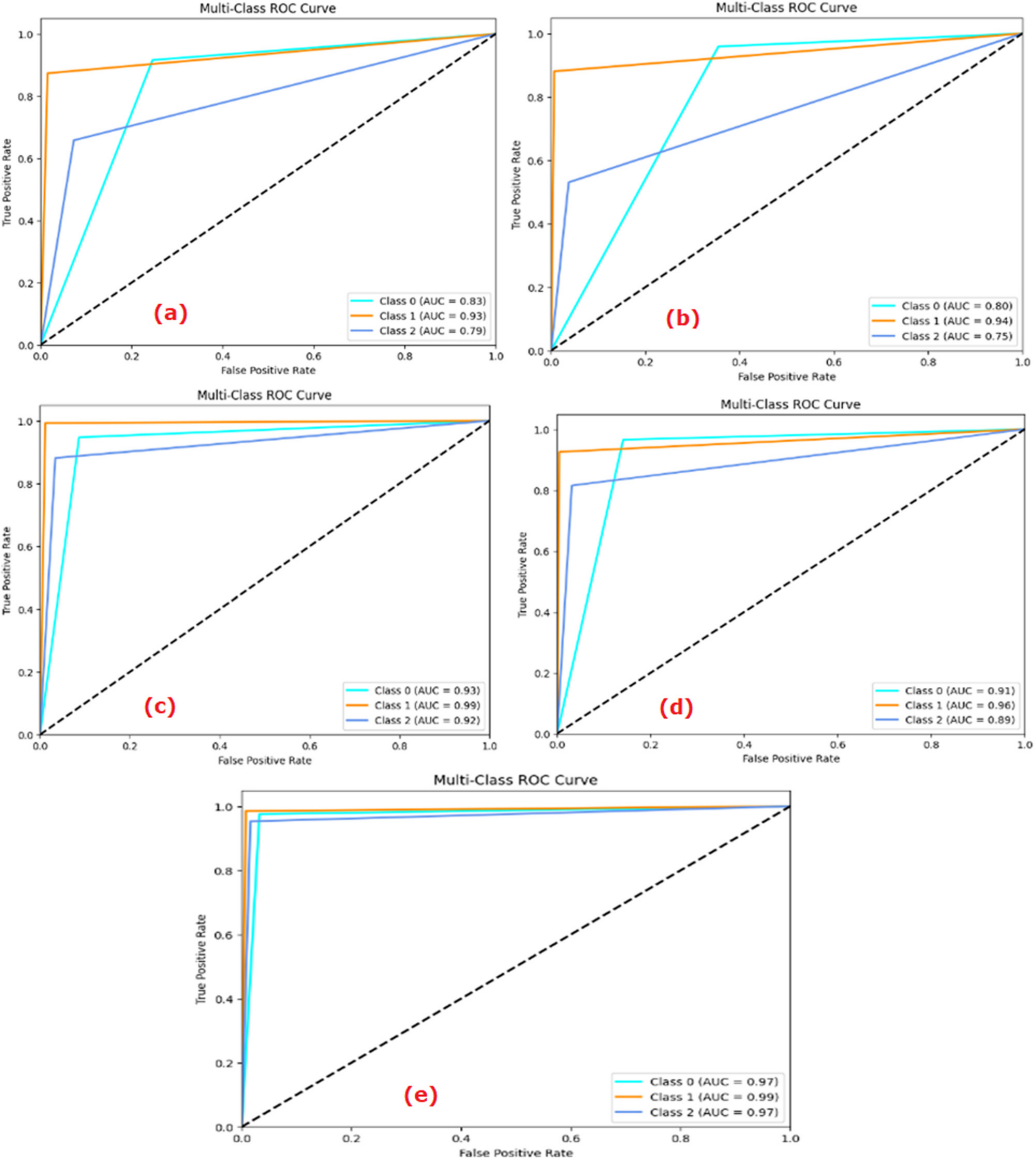
ROC curves: (a) LR, (b) *k*-NN, (c) VGG-19, (d) ViT, and (e) ConvMixer.

The values of the performance metrics parameters like accuracy, precision, recall, *F*1-score, and AUC are provided in Table 2. The AUC curve of each ML and DL model is shown in Figure 4. From the AUC curves (Figure 4), it was demonstrated that ConvMixer gave the highest AUC values for class 0 = 0.97, class 1 = 0.99, and class 2 = 0.97 compared to other ML/DL models for multiclass classification. The output of the multiclass classification of the ConvMixer model is shown in Figure 4. The accuracy obtained by the ConvMixer model was 97.09.

**Table 2:** ML-DL model’s performance evaluation for multiclass classification of CXRs

ML-DL model	Class type*	Precision	Recall	*F*1-score	AUC score	Accuracy
Performance evaluation of the two ML models						
Logistic regression	0	0.88	0.92	0.90	0.83	85.02
	1	0.85	0.87	0.86	0.93	
	2	0.74	0.66	0.70	0.79	
*k*-nearest neighbour	0	0.85	0.96	0.90	0.80	84.96
	1	0.93	0.88	0.90	0.94	
	2	0.82	0.53	0.64	0.75	
Performance evaluation of the three DL models						
VGG-19	0	0.96	0.95	0.95	0.93	93.53
	1	0.89	0.99	0.94	0.99	
	2	0.89	0.88	0.89	0.92	
ViT	0	0.93	0.97	0.95	0.91	92.61
	1	0.95	0.93	0.94	0.96	
	2	0.89	0.81	0.85	0.89	
ConvMixer	0	0.98	0.98	0.98	0.97	97.09
	1	0.93	0.99	0.96	0.99	
	2	0.95	0.95	0.95	0.97	

*Class 0: normal (control); Class 1: viral pneumonia; Class 2: COVID-19.

The ROC curve represented the ability of each model to diagnose at varying threshold levels. It is the curve between the true positive rate (no. of positive samples that are correctly predicted by an ML model) and the false positive rate (no. of actual negative samples that are incorrectly predicted as positive by the ML model). The ability of a model to accurately diagnose depends on the area covered by each ROC curve. More area meant more classification ability of the ML model. As observed in Figure 4, the AUC scores obtained for Class 0, Class 1, and Class 2 by the LR model were, respectively, 0.83, 0.93, and 0.79. The values similarly were 0.80, 0.94, and 0.75, respectively, in *k*-NN; 0.85, 0.91, and 0.82 in CNN; 0.93, 0.99, and 0.92 in VGG-19; 0.91, 0.96, and 0.89 in ViT; and 0.97, 0.99, and 0.97 in the ConvMixer models.

**Figure 5:**
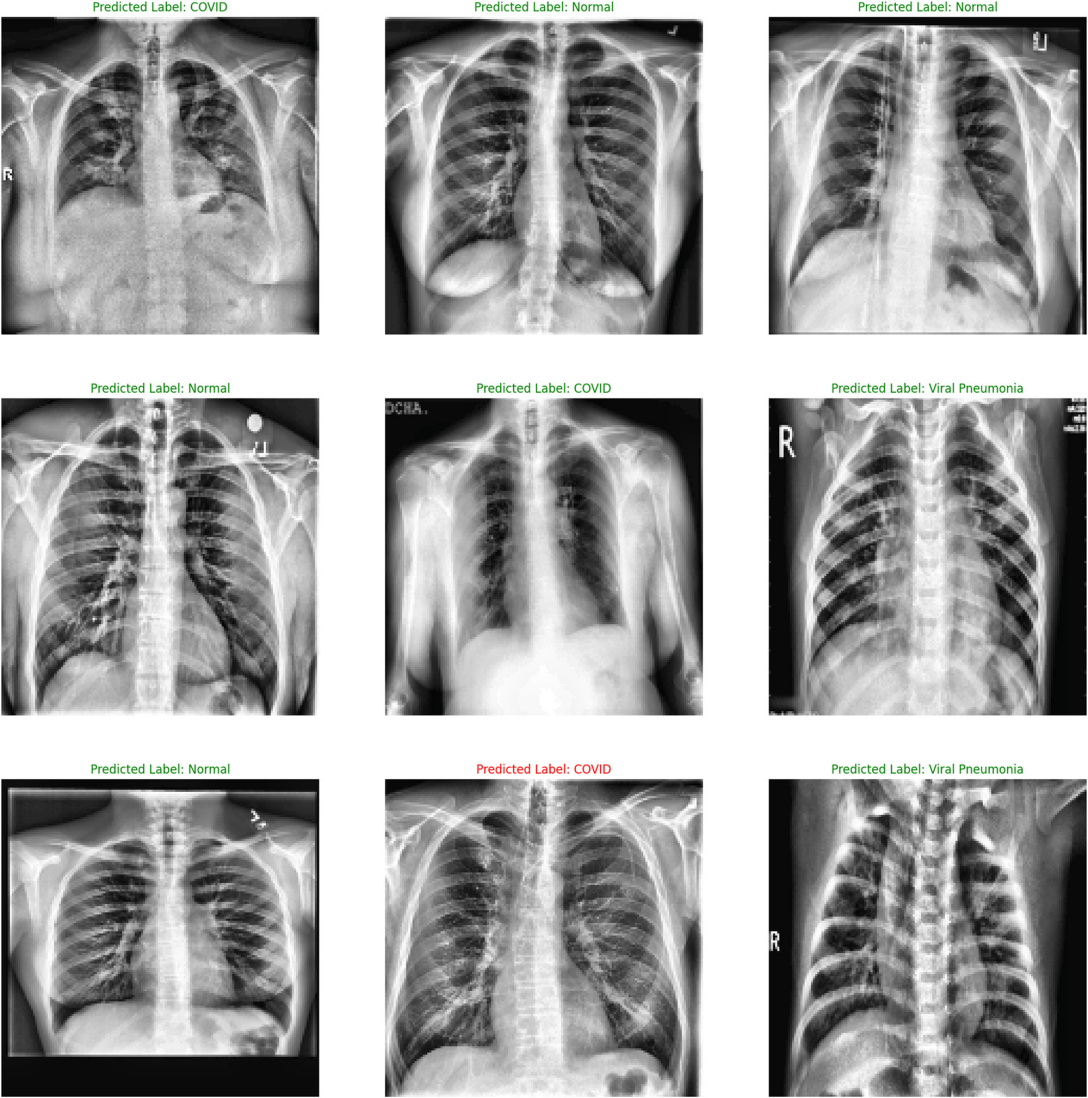
The normal, COVID, and viral pneumonia outputs from CXRs by ConvMixer.

**Figure 6:**
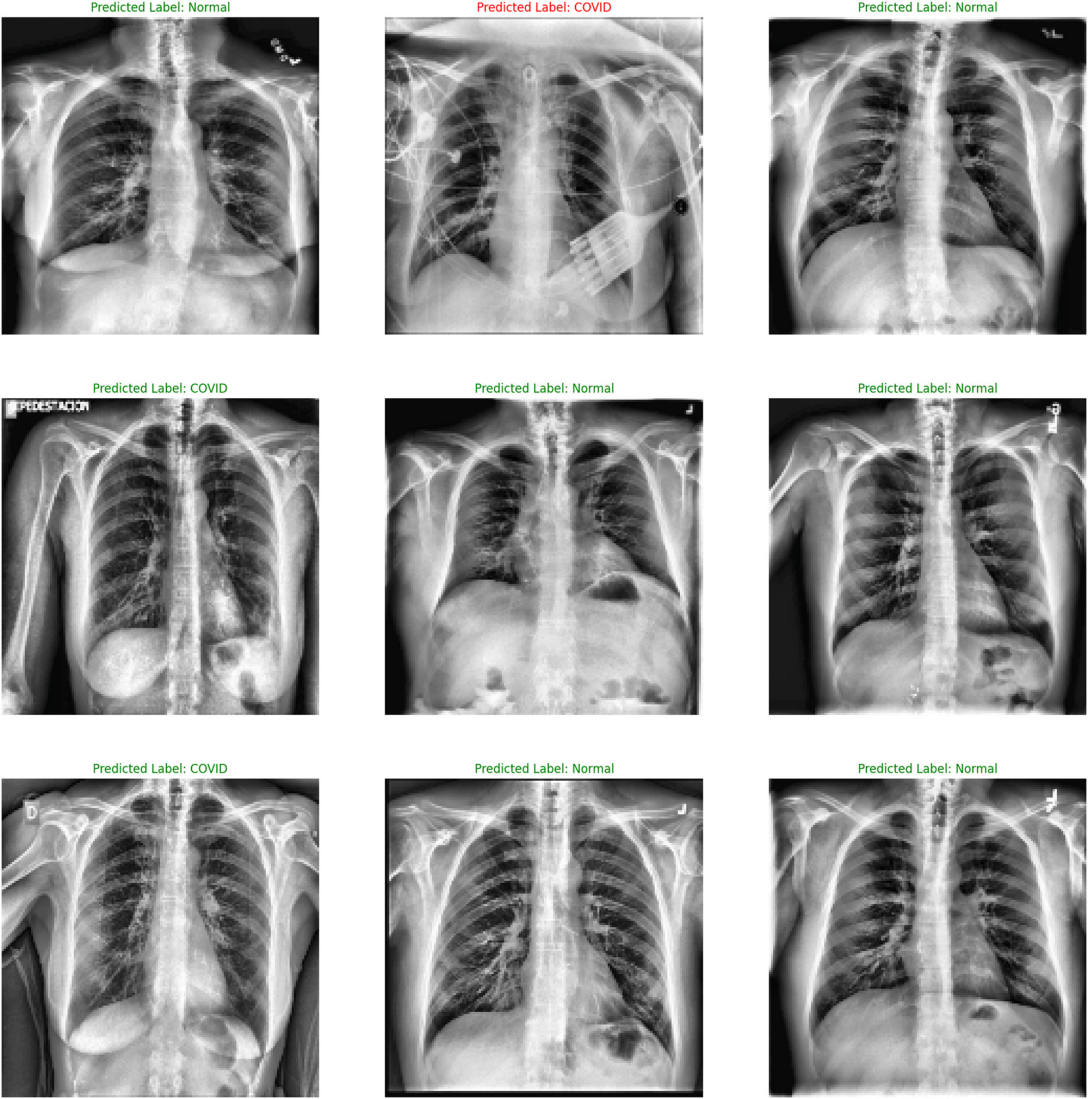
The output of the ConvMixer model for binary (COVID/normal) classification.

As seen from the output of the ConvMixer model (Figure 5), a normal CXR (left panel) portrayed clear lungs with no abnormal opacification. Bacterial pneumonia (middle set) exhibited a focal lobar consolidation (in the right upper lobe in this case), whereas viral pneumonia (right panel) set showed a distinct and more diffused “interstitial” pattern in both the lungs. The binary (COVID/normal) classification output of the ConvMixer model is shown in Figure 6.

The values of the performance parameters like precision, recall, *F*1-score, AUC, and accuracy in ML-DL models are provided in Table 3. The values obtained against the performance parameters showed that ConvMixer had the maximum accuracy (98%) and 0.97 AUC for COVID-19 infected lungs classification against the normal lungs.

**Table 3:** Performance comparison of the models for binary classification

ML-DL model	Class type*	Precision	Recall	*F*1-score	AUC score	Accuracy
Logistic regression	0	0.91	0.91	0.91	0.82	86.89
	1	0.75	0.75	0.75		
*k*-NN	0	0.87	0.97	0.91	0.77	86.53
	1	0.87	0.57	0.69		
VGG-19	0	0.98	0.95	0.96	0.94	94.71
	1	0.87	0.94	0.90		
ViT	0	0.96	0.98	0.97	0.93	95.87
	1	0.94	0.88	0.91		
ConvMixer	0	0.99	0.99	0.99	0.97	98.00
	1	0.96	0.96	0.96		

*Class 0: normal (control); Class 1: viral COVID-19.

**Figure 7:**
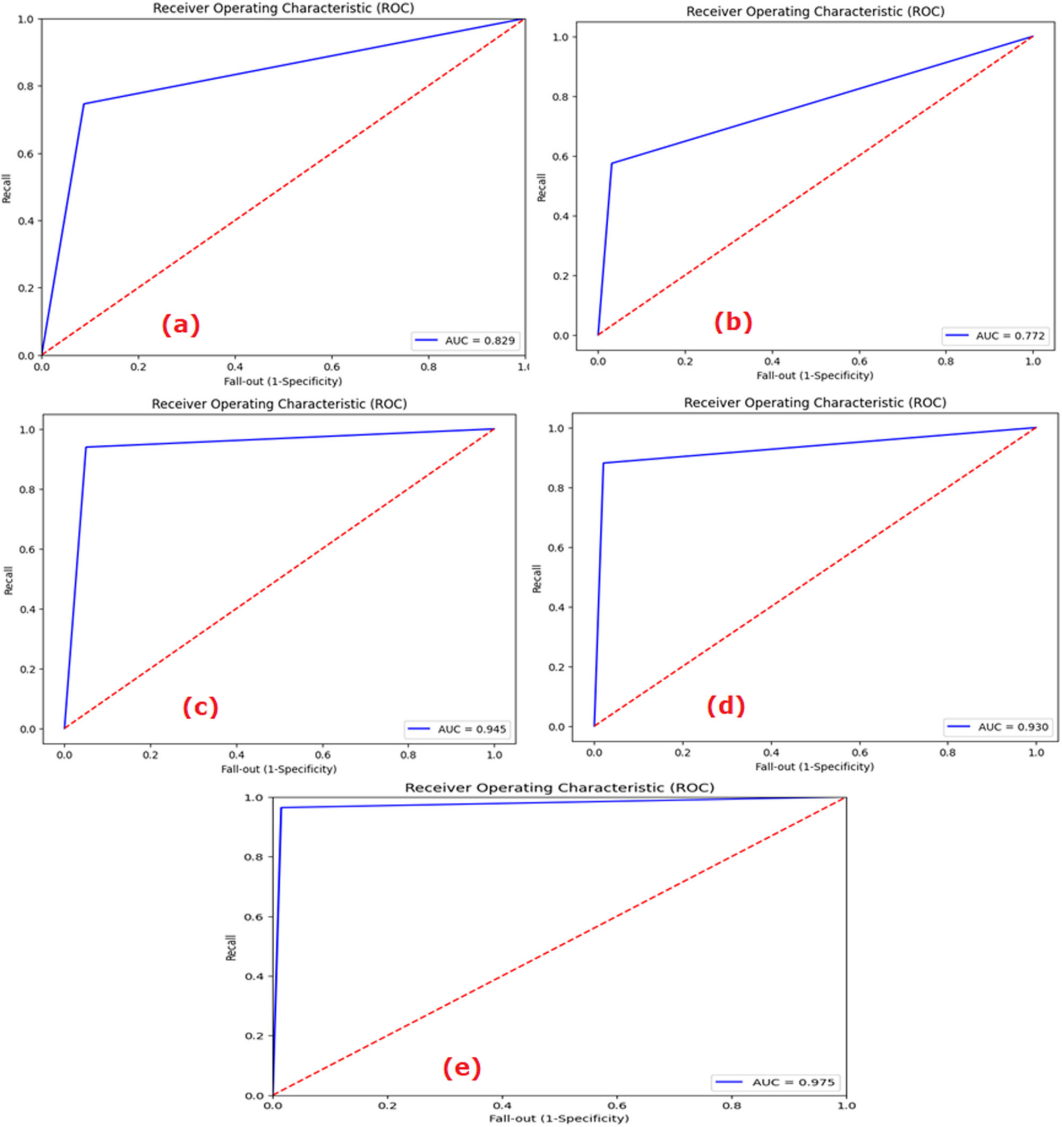
ROC curves of (a) LR, (b) *k*-NN, (c) VGG-19, (d) ViT, and (e) ConvMixer differentiating COVID-19-infected lungs from the normal ones.

The ROC curves of the models to diagnose COVID-19-infected lungs from normal lungs are shown in Figure 7.

The comparative performance values of the considered two ML and three DL models to diagnose and differentiate the bacterial and viral pneumonia against the control (normal) are detailed in Table 4.

**Table 4:** Comparative performance of the models to detect bacterial and viral pneumonia

ML-DL model	Class type*	Precision	Recall	*F*1-score	AUC score	Accuracy
Performance evaluation of the ML models						
Logistic regression	0	0.84	0.89	0.87	0.91	74.57
	1	0.75	0.81	0.78	0.79	
	2	0.59	0.47	0.52	0.68	
*K*-NN	0	0.88	0.91	0.90	0.93	76.27
	1	0.79	0.80	0.80	0.80	
	2	0.57	0.53	0.55	0.70	
Performance evaluation of the DL models						
VGG-19	0	0.94	0.91	0.92	0.94	77.13
	1	0.73	0.89	0.80	0.79	
	2	0.66	0.40	0.50	0.67	
ViT	0	0.94	0.94	0.94	0.96	76.62
	1	0.75	0.82	0.78	0.79	
	2	0.59	0.48	0.53	0.68	
ConvMixer	0	0.87	0.93	0.90	0.94	81.56
	1	0.79	0.89	0.84	0.84	
	2	0.79	0.55	0.65	0.75	

*Class 0: normal (control); Class 1: viral pneumonia; and Class 2: COVID-19.

**Figure 8:**
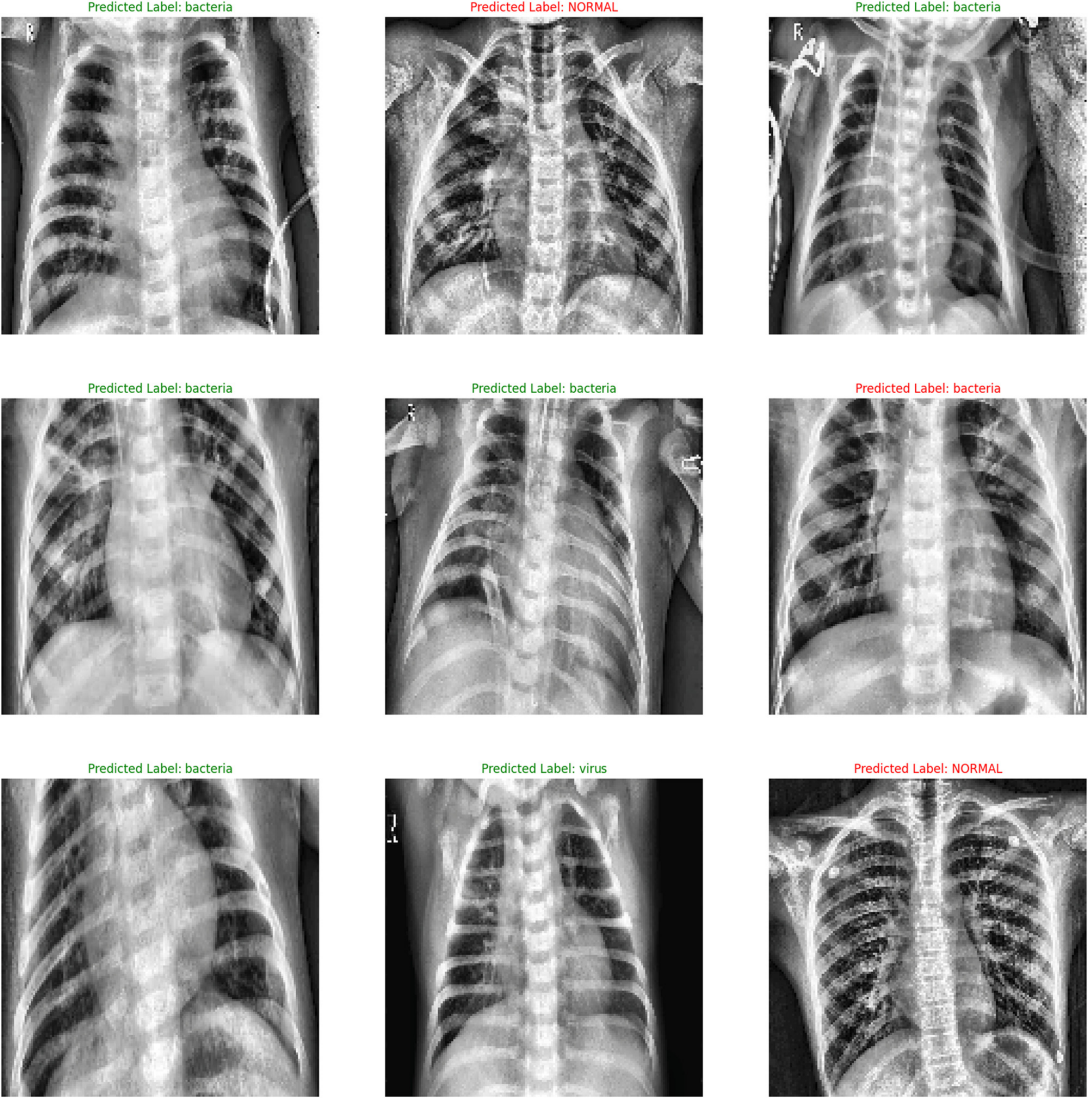
ConvMixer model outputs classifying viral pneumonia, bacterial pneumonia, and normal lung.

Figure 8 shows the classifiability of the various types of pneumonia (bacterial and viral) by ConvMixer from the CXR image database. From the output, it was observed that the normal CXR images (left panel) portrayed healthy lungs clearly without any abnormal opacification. The bacterial pneumonia (middle panel) typically exhibited a focal lobar consolidation, in this case in the right upper lobe (white arrows), and the viral pneumonia (right panel) was distinct with a more diffuse “interstitial” pattern in both the lungs.

**Figure 9:**
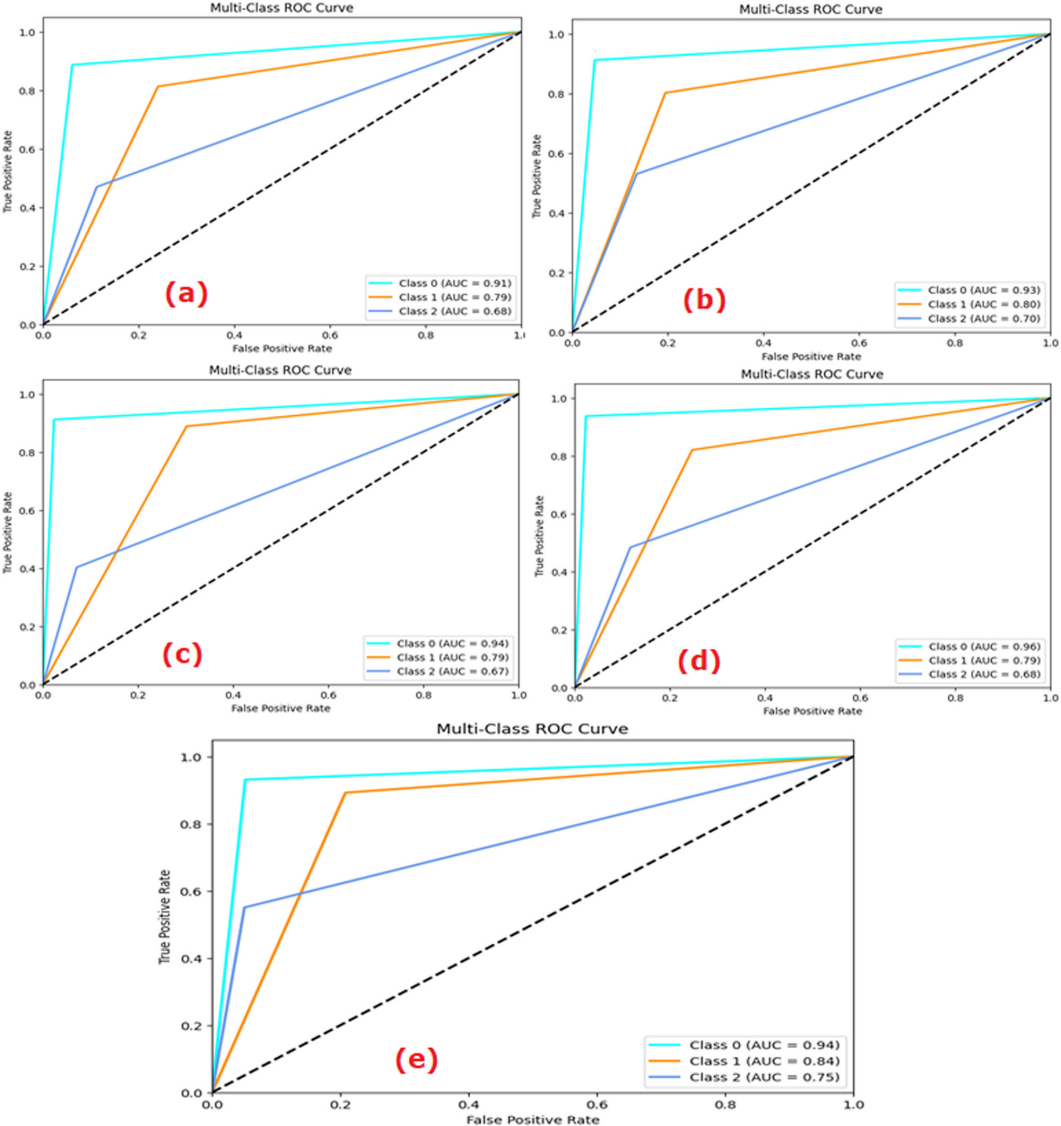
ROC of (a) LR, (b) *k*-NN, (c) VGG-19, (d) ViT, and (e) ConvMixer to detect normal, bacterial-infected, and viral-infected lungs.

Analysing the CXR images, the AUC scores obtained in the LR model for normal (Class 0), bacterial (Class 1), and viral (Class 2) pneumonia lungs were, respectively, 0.91, 0.79, and 0.68 (Figure 9). The figures similarly were 0.93 (Class 0), 0.80 (Class 1), and 0.70 (Class 2) in the *k*-NN model; 0.92 (Class 0), 0.79 (Class 1), and 0.88 (Class 2) in the CNN model; 0.94 (Class 0), 0.79 (Class 1), and 0.67 (Class 2) in the VGG-19 model; 0.96 (Class 0), 0.79 (Class 1), and 0.68 (Class 2) in the ViT model; and 0.94 (Class 0), 0.84 (Class 1), and 0.75 (Class 2) in the ConvMixer model.

The performance comparison of the models for pneumonia-affected lung and normal lung detection is given in Table 5. The maximum AUC and accuracy of 0.97 and 97.61% were obtained by the ConvMixer model for the classification of pneumonia-infected lungs from normal lungs, followed by the VGG-19 model with an AUC and accuracy of 0.95 and 96.75, respectively.

**Table 5:** Comparing the studied models’ performances to detect pneumonia-infected lungs

ML-DL model	Class type*	Precision	Recall	*F*1-score	AUC score	Accuracy
Logistic regression	0	0.88	0.76	0.84	0.87	92.32
	1	0.92	0.96	0.95		
*k*-NN	0	094	0.86	0.90	0.92	86.53
	1	0.95	0.98	0.96		
VGG-19	0	0.97	0.91	0.94	0.95	96.75
	1	0.97	0.99	0.98		
ViT	0	0.95	0.91	0.93	0.948	96.41
	1	0.97	0.98	0.98		
ConvMixer	0	0.96	0.95	0.96	0.97	98.0
	1	0.98	0.99	0.98		

*Class 0: normal (control); Class 1: pneumonia.

Figure S1 shows the pneumonia-infected lungs and normal lungs as detected by the ConvMixer model. The pneumonia classification AUC scores obtained by the LR, *k*-NN, CNN, VGG-19, ViT, and ConvMixer models, respectively, were 0.87, 0.92, 0.93, 0.95, 0.94, and 0.97 (Figure S2).

## 4 Discussion

The literature on the recently reported studies about COVID-19 case diagnosis using ML and DL approaches was surveyed for a greater and clearer understanding of the state of the affair. A few selected ones are discussed here. Kanakaprabha and Radha [27] used the CNN method to detect COVID-19 with 95% accuracy. They also detected viral and bacterial pneumonia with 91.46 and 80% accuracy, respectively. Sharma and Tiwari [28] classified COVID-19 and pneumonia using the CXR image database with 94% accuracy. Yaseliani et al. [29] used an ensemble classifier LR and support vector machine with radial basis function to detect pneumonia at 98.55% accuracy. Arias-Garzón et al. [30] achieved 97% detection accuracy for COVID-19 using VGG19 and UNET approaches. Jain et al. [31] used CNN and transfer learning approaches to detect pneumonia from X-ray images and obtained 93.31% accuracy in the CNN model. Moreover, Zhang et al. [32] developed a VGG-based architecture to detect pneumonia and reported accuracy of 96.06, 0.99, 94.408, 90.823, and 92.851%, respectively, for AUC, precision, recall, and *F*1-score. Mabrouk et al. [33] obtained 93.91% accuracy and 93.88% *F*1-score employing ensemble learning approaches (Densenet161, mobilenetv2, and ViT). Hashmi et al. [34] proposed the Resnet 50 model to detect pneumonia with 98.14% test accuracy. Recently, Ibrahim et al. [35] used a pre-trained AlexNet model to classify pneumonia and COVID-19 from CXR images with 93.42% accuracy. Makarovskikh and his group [36] classified SARS-CoV-2 positive and normal images using the Densenet121 model with 98.97% accuracy. Our group also developed an IoT-based COVID-19 detection protocol using ML models and obtained 98% accuracy with the *k*-NN model [21]. Recently, we have also classified COVID-19 and pneumonia from the CXR images by using DL models and obtained 97% accuracy for VGG16 [24].

We have also compared the performance of various models and the state-of-the-art outputs in other similar reported studies, as detailed in Table 6. Using the ensemble model (ResNet18, AlexNet, Inception v3, GoogleNet, and DenseNet121), Chouhan et al. [37] obtained 96.4% accuracy in detecting pneumonia and COVID-19-infected lungs. Using a pre-trained deep CNN model, Liang and Zheng [38] reported 96.7% accuracy in detecting pneumonia in children. The results of the test dataset showed that the recall rate of the method was 96.7% in classifying children with pneumonia, and the *F*1 score was 92.7%. Using the VGG-16 model, Brunese et al. [39] obtained 96% accuracy, 98% specificity, and 96% sensitivity using the CXR image technique to detect COVID-19 and other pulmonary diseases. Using the CNN model to detect pneumonia from CXR images, Stephen et al. [40] obtained 95% accuracy.

**Table 6:** Comparison of the observed performances in the study with other reported studies

Literature	Dataset	% accuracy	Class	% specificity	% sensitivity	Reference no.
Chouhan et al. (2020)	Public	96.39	3	—	—	[37]
Liang and Zheng (2020)	Public	90	2	—	—	[38]
Brunese et al. (2020)	Public	96	2	98	96	[39]
Stephen et al. (2019)	Public	95	2	—	—	[40]
Wang et al. (2021)	Public	89.5	3	0.88	0.87	[41]
Jaiswal et al. (2020)	Public	96.25	2	96.21	96.29	[42]
Apostolopoulos and Mpesiana (2020)	Public	96.78	3	98.66	96.44	[43]
**Proposed model**	**Public**	**97.09**	**3**	**98.4**	**97.4**	**This study**

Using Inception v3 model to detect COVID-19 and pneumonia-infected lungs, Wang et al. [41] demonstrated 89.5% accuracy, 0.88% specificity, and 0.87% sensitivity. Recently, Jaiswal et al. [42] reported 96.25% accuracy, 96.21% precision, and 96.29% recall using the DenseNet 201 model. Furthermore, Apostolopoulos and Mpesiana [43] obtained 96.78% accuracy, 98.66% specificity, and 96.46 sensitivity using transfer learning approaches to analyse pneumonia, COVID-19, and normal lung CXRs. ConvMixer, the proposed model under the present study, returned the best accuracies (97% for multiclass and 98% for binary class, with combined augmentation techniques) as compared to other similar stand-alone state-of-the-art models.

From the results of the present study, it was observed that the ConvMixer model returned the best accuracies (97% for multiclass and 98% for binary class) through combined augmentation in comparison to other test models. As the proposed model showed greater efficiency in terms of accuracy, specificity, and sensitivity it was recommended to observe to investigate the real-life performance of ConvMixer. Robust evaluation of the efficacy of ConvMixer with its practical utility will aid and contribute significantly to the healthcare sector not only for the benefit of the less-endowed low-income countries but also others. We believe that this will solve the critical challenges in medical diagnosis. It is reported that the SARS-CoV-2 virus was capable of infecting domestic and wild animals, too, through spillback infections [44]. Thus, the present investigation on the predictive models shall help diagnosis and well-being in both humans and animals.

## 5 Limitations of the study

Although numerous important datasets are freely available online, however, such datasets on CXR images of COVID-19 patients are limited. To overcome this difficulty, only two reliable datasets of CXR images were chosen for investigations in the present study. Both datasets are smaller and have limited COVID-19-related data. Further, the used datasets are reliable but imbalanced. The used datasets had a total of four categories (normal healthy, bacterial, viral, and COVID-19 infected) data. The performance of the proposed model (ConvMixer) must be evaluated to implement in real-life situations by using larger and more categorised datasets.

Some images are of different standards, quality, and sizes, and hence it is highly essential to store the images following the standard operating procedures to allow researchers to utilise the data freely for better image classification. The CXRs are less sensitive than CT scans and may generate false predictions in early and mild cases [45,46]. Therefore, a CT scan could seemingly be more reliable for COVID-19 diagnosis. However, such datasets are limited due to high cost, high radiation dose, and limited resource availability. It is also recommended that the efficacy of the proposed ConvMixer model could be evaluated on CT scan image datasets for further validation.

## 6 Conclusion

The present work aimed to differentiate and classify healthy lungs (the control) from the COVID-19 and pneumonia-infected lungs based on the CXR images using computer-aided ML and DL models. The viral or bacterial pneumonia-infected lung subtypes were also detected. The ConvMixer model returned detection accuracies of 97.01 and 98% in detecting COVID-19-infected lungs from normal lungs. This model detected bacterial and viral pneumonia variants with 81.72 and 98% accuracies, respectively. After evaluating and comparing five ML/DL models to identify, classify, and categorise COVID-19 and other pneumonia infection forms from the CXR images, ConvMixer is suggested as the best model for COVID-19 detection. The suggested model could accomplish tasks involving three classes and binary classification with overall 97.01, 81.72, and 98% accuracy rates, respectively, for normal, COVID-19, and other pneumonia cases.

The suggested model could be an effective tool to implement in resource-less nations for speedy and reliable diagnoses of the cases affected by COVID-19 or similar infections in the pulmonary and respiratory systems. It will also address the issue of the lack of detection or diagnostic resources in such regions. The suggested ConvMixer network could effectively detect COVID-19 and various other pneumonia using CXR images, which would help radiologists make a timely and accurate diagnosis. The accuracies of these models could further be augmented for diagnosis by using the ensemble techniques.

## Supplementary Material

Supplementary material
