# Supplementary material for: A comparative analysis of the binary and multiclass classified chest X-ray images of pneumonia and COVID-19 with ML and DL models
Source: Open Med (Wars). 2025 Feb 4;20(1):20241110. doi: 10.1515/med-2024-1110 (PMC11806240; doi:10.1515/med-2024-1110)
Supplement: Supplementary material [file med-2024-1110-sm.pdf]

Supplementary material

Table S1: The layer-wise structure of VGG-19

Layer (type)	Output shape	Number of parameters
input_1 (InputLayer)	(150, 150, 3)	0
block1_conv1 (Conv2D)	(150, 150, 64)	1,792
block1_conv2 (Conv2D)	(150, 150, 64)	36,928
block1_pool (MaxPooling2D)	(75, 75, 64)	0
block2_conv1 (Conv2D)	(75, 75, 128)	73,856
block2_conv2 (Conv2D)	(75, 75, 128)	147,584
block2_pool (MaxPooling2D)	(37, 37, 128)	0
block3_conv1 (Conv2D)	(37, 37, 256)	295,168
block3_conv2 (Conv2D)	(37, 37, 256)	590,080
block3_conv3 (Conv2D)	(37, 37, 256)	590,080
block3_conv4 (Conv2D)	(37, 37, 256)	590,080
block3_pool (MaxPooling2D)	(18, 18, 256)	0
block4_conv1 (Conv2D)	(18, 18, 512)	1,180,160
block4_conv2 (Conv2D)	(18, 18, 512)	2,359,808
block4_conv3 (Conv2D)	(18, 18, 512)	2,359,808
block4_conv4 (Conv2D)	(18, 18, 512)	2,359,808
block4_pool (MaxPooling2D)	(9, 9, 512)	0
block5_conv1 (Conv2D)	(9, 9, 512)	2,359,808
block5_conv2 (Conv2D)	(9, 9, 512)	2,359,808
block5_conv3 (Conv2D)	(9, 9, 512)	2,359,808
block5_conv4 (Conv2D)	(9, 9, 512)	2,359,808
block5_pool (MaxPooling2D)	(4, 4, 512)	0

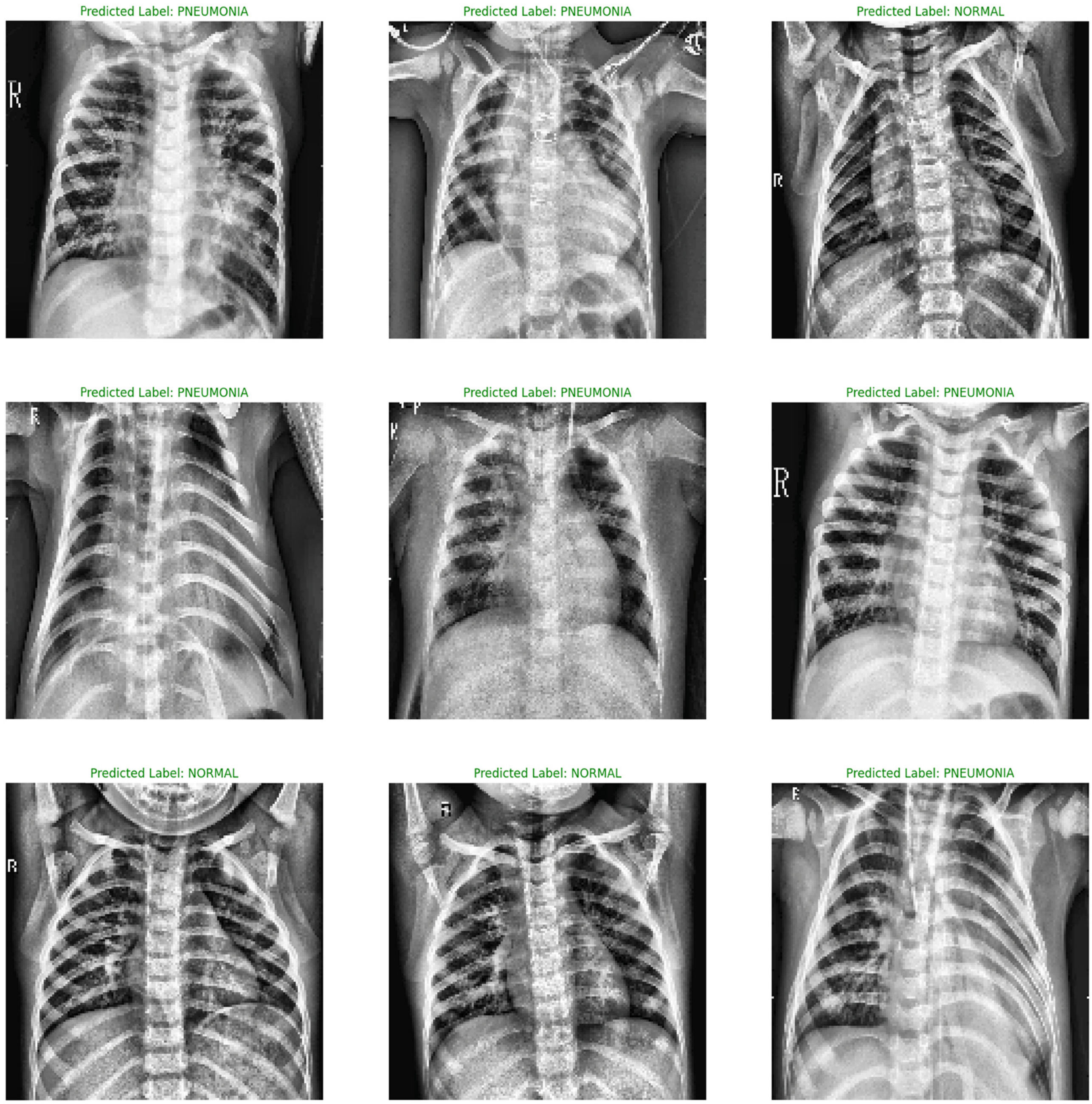

Figure S1: Compared image outputs of pneumonia-infected and normal lungs by ConvMixer.

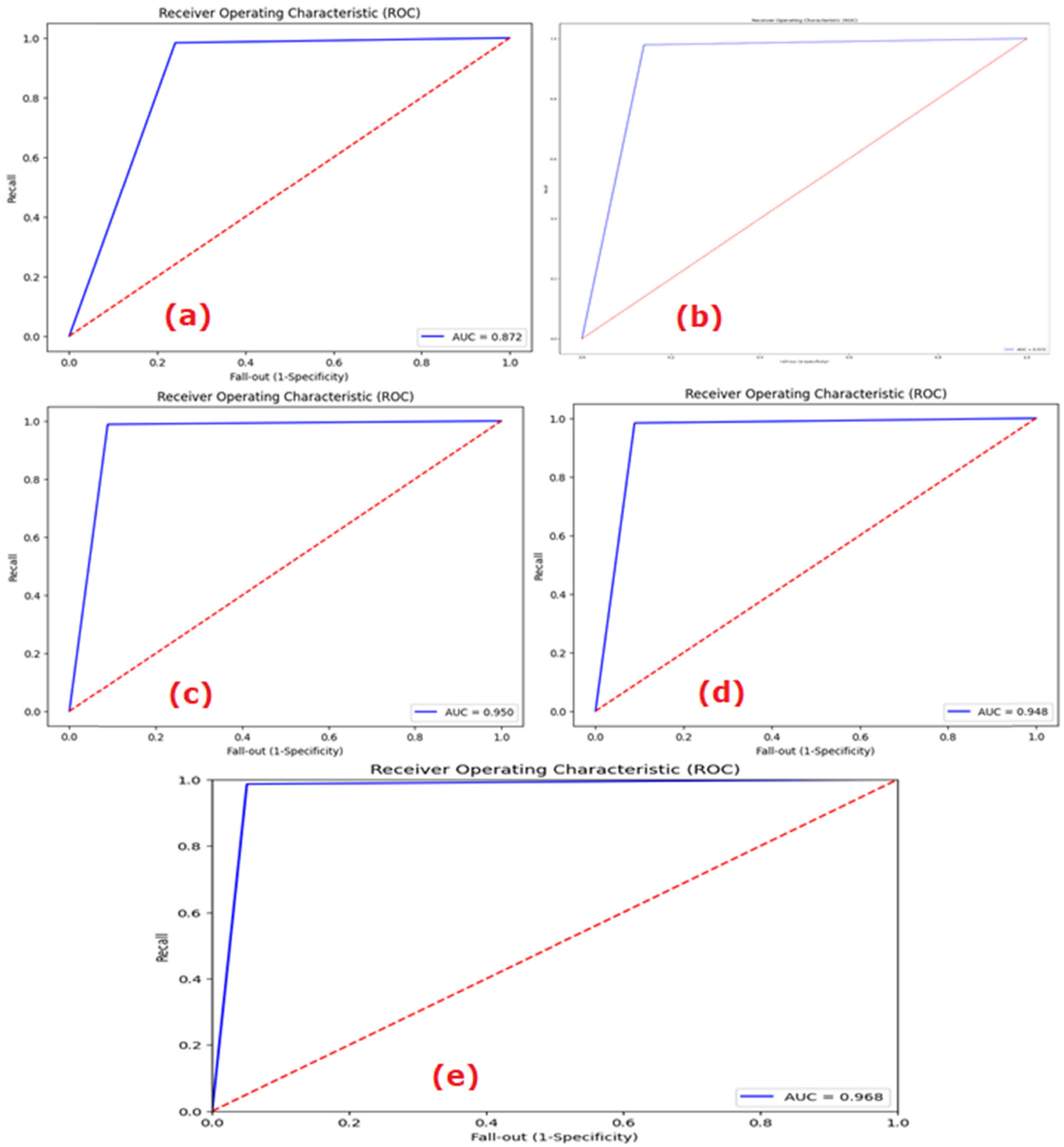

Figure S2: The ROCs of (a) LR, (b) *k*-NN, (c) VGG-19, (d) ViT and (e) ConvMixer models.
